# Decreased Resting-state fMRI Local Coherence in Schizophrenia Patients with Poor Long-term Outcome

**DOI:** 10.1192/j.eurpsy.2022.638

**Published:** 2022-09-01

**Authors:** Y. Panikratova, E. Abdullina, D. Tikhonov, V. Kaleda, I. Lebedeva

**Affiliations:** 1 Mental Health Research Center, Laboratory Of Neuroimaging And Multimodal Analysis, Moscow, Russian Federation; 2 Mental Health Research Center, Department Of Youth Psychiatry, Moscow, Russian Federation

**Keywords:** resting-state fMRI, Local Correlation, outcome, schizophrénia

## Abstract

**Introduction:**

Schizophrenia is heterogeneous in terms of symptoms and outcome, but neurobiology of this heterogeneity is not well-studied. Local correlation analysis of fMRI data provides a measure of local coherence, i.e., average correlation between BOLD-signal in a voxel and its neighbours. Local correlation is a promising approach, and it seems important to find links between local brain coherence and schizophrenia outcome.

**Objectives:**

We aimed to compare brain local coherence between schizophrenia patients with varied long-term outcomes and healthy controls (HC).

**Methods:**

Patients with chronic schizophrenia spectrum disorders (37 males, mean age 41.5±5.5) and HC (17 males, mean age 38±7.7) underwent resting-state fMRI (3T). Cluster analysis based on PANSS and PSP allowed us to allocate patients into two subgroups (N = 13/24). The second subgroup had significantly more marked negative and general psychopathology symptoms and worse functioning than the first subgroup. Local coherence in the brain was compared between clinical subgroups and HC (ANOVA, *p*<.001 voxelwise, *p[FDR]*<.05 clusterwise).

**Results:**

Local coherence in the paracingulate gyri bilaterally ({-2; 58; 14}; 2712 mm^3^) differentiated the groups. *Post hoc* analysis revealed decreased local coherence in the subgroup with poorer outcome compared to HC, along with the absence of differences between the subgroup with better outcome and HC. There were no differences between clinical subgroups.

**Conclusions:**

Hypoactivity of the cingulate cortex is related to negative symptoms (Bersani et al., 2014). Their severity, in turn, is strongly associated with outcome. Thus, local coherence in the cingulate cortex may be one of the factors which underlie outcome heterogeneity.

**Disclosure:**

No significant relationships.

